# Reducing burden from respiratory infections in refugees and immigrants: a systematic review of interventions in OECD, EU, EEA and EU-applicant countries

**DOI:** 10.1186/s12879-021-06474-0

**Published:** 2021-08-26

**Authors:** Jan-Frederic Lambert, Katarina Stete, James Balmford, Annabelle Bockey, Winfried Kern, Siegbert Rieg, Martin Boeker, Berit Lange

**Affiliations:** 1 Division of Infectious Diseases, Department of Medicine II, Medical Center and Faculty of Medicine, University of Freiburg, Hugstetter Straße 55, 79106 Freiburg im Breisgau, DE Germany; 2 Institute of Medical Biometry and Statistics, Faculty of Medicine and Medical Center, University of Freiburg, Freiburg im Breisgau, Germany; 3 Department of Epidemiology, Helmholtz Centre for Infection Research, Inhoffenstr.7, 38124 Braunschweig, DE Germany

**Keywords:** Respiratory infections, Vaccination, Hygiene, Health literacy, Early interventions, Refugees, Migrants

## Abstract

**Background:**

Respiratory diseases are a major reason for refugees and other immigrants seeking health care in countries of arrival. The burden of respiratory diseases in refugees is exacerbated by sometimes poor living conditions characterised by crowding in mass accommodations and basic living portals. The lack of synthesised evidence and guideline-relevant information to reduce morbidity and mortality from respiratory infections endangers this population.

**Methods:**

A systematic review of all controlled and observational studies assessing interventions targeting the treatment, diagnosis and management of respiratory infections in refugees and immigrants in OECD, EU, EEA and EU-applicant countries published between 2000 and 2019 in MEDLINE, CINAHL, PSYNDEX and the Web of Science.

**Results:**

Nine of 5779 identified unique records met our eligibility criteria. Seven studies reported an increase in vaccine coverage from 2 to 52% after educational multilingual interventions for respiratory-related childhood diseases (4 studies) and for influenza (5 studies). There was limited evidence in one study that hand sanitiser reduced rates of upper respiratory infections and when provided together with face masks also the rates of influenza-like-illness in a hard to reach migrant neighbourhood. In outbreak situations of vaccine-preventable diseases, secondary cases and outbreak hazards were reduced by general vaccination strategies early after arrival but not by serological testing after exposure (1 study). We identified evidence gaps regarding interventions assessing housing standards, reducing burden of bacterial pneumonia and implementation of operational standards in refugee care and reception centres.

**Conclusions:**

Multilingual health literacy interventions should be considered to increase uptake of vaccinations in refugees and immigrants. Immediate vaccinations upon arrival at refugee housings may reduce secondary infections and outbreaks. Well-designed controlled studies on housing and operational standards in refugee and immigrant populations early after arrival as well as adequate ways to gain informed consent for early vaccinations in mass housings is required to inform guidelines.

**Supplementary Information:**

The online version contains supplementary material available at 10.1186/s12879-021-06474-0.

## Background

Worldwide, more than 60 million people were fleeing from war, violence, climate catastrophes, human rights violations and other circumstances at the end of 2017 [1]. Difficult living conditions, the context of having fled from hardship and cultural and language barriers can limit health care access and uptake, as well as result in missed opportunities for prevention, vaccination and treatment [2, 3].

Respiratory infections are a major reason why refugees and immigrants seek healthcare [4, 5]. There is agreement that refugees and asylum seekers are not a notable source of transmission of respiratory disease to the general population [5–7]. Refugees and asylum seekers often are exposed to difficult living and housing conditions in sometimes improvised camps, shelters, and reception centres especially during the period of travel after forced migration, but also during the first months after arrival. In some communities not only refugees but also other immigrants may experience similar conditions due to crowded households, missing financial resources, unsanitary housings, and a lack of hygiene. Whenever talking in general about this population we therefore use the term “refugees and immigrants’’. However, due to these difficult living and housing conditions refugees and immigrants are not only at higher risk of respiratory infections [8], but also at higher risk of critical illness from vaccine preventable diseases [9] than non-migrant populations. This makes respiratory tract-related infections and vaccine-preventable diseases particularly important health issues in this population [4, 4, 8, 10–12].

To our knowledge no specific guidelines on reducing the overall burden of respiratory infectious diseases in refugees and immigrants exists. Instead national guidelines mostly focus on vaccine-preventable diseases not always specifically mentioning refugees and immigrants. Also, there is little consensus across different guidelines on which strategies should be applied [13–21]. There is a lack of specific guidelines targeting the management of respiratory infections as well as guidelines specifically aiming on refugee and immigrant populations (see Table 1).

**Table 1:** National and international guidelines with recommendations regarding infectious diseases, including respiratory infectious diseases

Guideline		Main recommendations regarding particular issues			
Region/Country	Guideline	Vaccinations	Screening or other diagnostic assessment	Health-literacy	Housing
Europe[21]	European Centers for Disease Control and Prevention (ECDC) / WHO regional office for Europe www.ecdc.europa.eu	Country specific vaccination schedule should be implemented for all refugees/asylum seekers/migrants staying for one week or more Main priority should be MMR and Polio vaccines Vaccination should not be implemented at border crossings unless in the presence of an outbreak	Regarding less of their legal status equitable and non-discriminatory access to essential health care services with preventive and curative interventions should be granted to all refugees and asylum seekers All member countries should be prepared at any time to epidemiologically assess and adequately react to any outbreak situation	None	Overcrowding should be avoided (not giving numbers) Clean sanitary institutions and clothing should be available
Switzerland[20]	Schweizerische Gesellschaft für Infektionskrankheiten www.Sginf.ch	Following the same vaccination schedule as for indigenous previously unimmunised adults/children Vaccination only valid when written documentation is available	All refugee children under 5 y.o. Should be screened for TB using tuberculin skin test regardless of BCG vaccination status and country of origin All adults and children > 5 y.o. Should be screened for TB only in case of symptoms using IGRA or TST on Syphilis Screening for all children < 2 y.o. And all juvenile refugees 12–15 y.o	None	None
Germany[18, 19]	Robertkochinstitut www.rki.de	Documentation of vaccination status Valid documentation leads to enrolment of national vaccination schedule for all residents Invalid documentation leads to accomplishment of separate minimum vaccination schedule All refugees should be vaccinated against influenza not only high risk population	Examination not focussing on personal health status but on outbreak prevention in the first line General anamnesis focussing on infectious diseases Full body examination including vital parameters focussing on infectious diseases and rashes Screening for active lung TB by x-ray (exceptions for pregnant women and children under 15 y.o	None	None
Australia[13]	Australian Government—Departement of Health www.health.gov.au	Vaccination status of refugees is not routinely assessed, differing by states All refugees arriving to Australia should receive vaccination catch-up vaccinations Pre-departure vaccinations and valid documentations of previous vaccinations should be considered and all vaccinations to children under 7 y.o. Should be reported to ACIR (Australian Childhood Immunisation Register)	In all arriving people with refugee-like background a general assessment regarding HIV, disability, strongyloides serology and other should be conducted Some assessments like syphilis serology, vitamin- or ferritin status should be conducted by individual risk Some assessments like Malaria status, Hepatitis C and Schistosoma serology should be conducted by respective country of origin	None	None
USA[14]	Centers for Disease Control and prevention www.cdc.gov	Vaccination only valid when written documentation is available Overseas vaccination program implemented by registered physicians before departure to US consisting of two doses of several vaccines with 1–2 months of time in between -post-arrival after assessment of immunization status an age-adjusted vaccination schedule will be implemented. Serologic testing may be used for evaluating vaccination-status	If possible medical examination by special physicians will be implemented before departure from overseas. Except from vaccination status assessment there will be also presumptive treatment with albendazole enrolled Broad medical examination by special physicians implemented during the first months after arrival in the US. Physical status as well as nutritional status, lead-test, laboratory testing and age adjusted TB screening will be assessed. Vaccination coverage is assessed and records from former overseas examination will be checked and completed	None	None
Canada[16]	National Advisory Committee on Immunization (NACI) Committee to Advise on Tropical Medicine and Travel (CATMAT) https://www.canada.ca/en/public-health/services/canadian-immunization-guide.html	Vaccination status is assessed prior to arrival with only documented proof of vaccination accepted as valid Catch-up schedule is oriented to country of origin and age of individual -MMR and Varicella vaccination should not be given in case of suspected active tuberculosis	Before arriving in Canada refugee claimants are enrolled in Immigration Medical Examinations TB-screening using TST is implemented to migrants from high-risk countries Laboratory examinations are implemented after arrival in Canada checking for full blood cell count, sickle cell status, HIV status (when coming from high-burden countries) and other	None	None
United Kingdom[17]	National Institute for Health Care Excellence https://www.gov.uk/government/collections/migrant-health-guide-countries-a-to-z	Vaccination status of refugees residing in the UK should always be assessed by physicians. Age adjusted national catch-up-schedule is implemented	Existing assessment guidelines for many different countries: every country of origin has its respective schedule for migrant health Newly arriving migrants are evaluated by assessing their psycho-social status, sexual-behaviour, ethinicity and risk of communicable diseases, as well as personal health issues such as hearing, seeing and risk factors for chronic diseases LTBI screening is implemented for all immigrants having spent significant time or being born in high-risk areas using TST or IGRA -active TB screening is implemented to all immigrants migrating from high incidence countries using chest x-ray	Newly arriving migrants should be informed how the NHS works and how it differs to health systems they are used to	None

Several recent reviews have criticised national screening guidelines as too restrictive, focussed on single diseases [22] and failing to cover the targeted populations [4, 22, 23], failing to provide information on how to improve screening and treatment coverage and completion [23], and being insufficiently based on evidence and evidence synthesis [24] (see Table 2). Previous studies of refugee and immigrant populations have mainly been concerned with screening and treatment strategies, as well as the cost-effectiveness of tuberculosis prevention in immigrants from high to lower-incidence regions [25–27]. Despite prevalence assessments of disease spectrums in migrant and refugee health and evaluations of implemented screening strategies[4, 5, 8], effective strategies and recommendations to reduce the burden of respiratory infections are lacking [9, 24, 28–30]. Healthcare providers often have insufficient training and are confronted with specific ethical, institutional and cultural issues as well as unfamiliar spectrums of diseases and symptoms [31–33]. Overviews and syntheses of evidence regarding effective interventions in refugees and immigrants are scarce, especially in industrial nations [24, 34, 35]. Yet evidence is urgently needed to inform recommendations for treatment, diagnostics, screening and effective prevention of infectious diseases [22, 28] in national and international guidelines.

**Table 2:** Previous or ongoing systematic reviews regarding infectious diseases and respiratory infections in refugees and asylum seekers

Authors (year)	Name of review	N references	Population	Infection(s)	Intervention(s) /exposure(s)	Main findings
Dasgupta, et al. (2005) [25]	Cost-effectiveness of tuberculosis control strategies among immigrants and refugees	72	Immigrants, refugees from high to low incidence-countries	Tuberculosis	All TB- related diagnostics*	1) Previously used chest-x-ray has minimal impact 2) Ideal control strategy would be global investment in high-incidence countries 3) Cell-mediated strategies are expensive and were not evaluated for screening purposes
Aldridge et al. (2014) [26]	Pre-entry screening programmes for tuberculosis in migrants to low-incidence countries	15	Migrants to low-incidence-countries	Tuberculosis	Pre-entry-screening (all TB- related diagnostics*)	1) Biggest yield for culture- and smear-based screening for individuals from high-incidence-countries 2) Ideal control strategy would be domestic returns for Investment in tuberculosis control programs overseas
Campbell et al. (2015) [27]	A systematic review on TST and IGRA tests used for diagnosis of LTBI in immigrants	51	Immigrants	Tuberculosis	TST and IGRA in low-incidence countries	1) TST and IGRA present similar sensitivity and specificity for active TB–IGRA may be preferred in immigrants 2) positive test prevalence was lower for individuals < 18 years old and individuals from low-incidence countries
De-vries et al. (2017) [41]	Barriers and facilitators to the uptake of tuberculosis diagnostic and treatment services by hard-to-reach	12	Hard-to-reach (mainly migrants)	Tuberculosis	Risk-factors for limited uptake of TB treatment and diagnostic	1) Tuberculosis-related Stigmatisation was perceived as a major barrier 2) Institutional barriers main factors for delay to diagnosis 3) No strong evidence on facilitators found 4) Cultural and language barriers main factors for health-care providers
Heuvelings et al. (2017) [29]	Effectiveness of interventions for diagnosis and treatment of tuberculosis in hard-to-reach populations in countries of low and medium tuberculosis incidence	19	Hard-to-reach	Tuberculosis	Treatment of active TB in OECD, EU, EEA and EU-applicant countries	1) Mobile chest-x ray units are an effective and easy way of diagnosing active TB, because of poor follow-up in this population 2) Active referral to TB clinics has been shown to be effective in migrants for the uptake of treatment 3) Community dot by non-family members seem to be most effective, some contradictions 4) Incentives are a valuable intervention to increase uptake of screening, diagnosis and adherence to treatment in homeless people and drug abusers
Bellos et al. (2010) [8]	The burden of acute respiratory infections in crisis-affected populations	36	Health-crises affected populations	Acute respiratory infections	Affected by health crises	1) High burden of ARI even increases during crises 2) Older children should be more integrated in vaccination strategies 3) More resources should be invested for ARI prevention and control
Bozorgmehr et al. (2017) [24]	Infectious disease screening in asylum seekers—range, coverage and economic evaluation in Germany, 2015	n.a	Refugees	Screened infectious diseases	Health screening implemented by German states	1) Newly arrived refugees are mainly affected by screening for active TB, STI and stool parasites 2) Expenses for screening using private fees could be 30% higher 3) High costs in diseases with low yield argue for more evidence-based approaches in screening methods
Crocker-buque et al. (2017) [79]	Immunization, urbanization and slums—a systematic review of factors and interventions	63	Hard-to-reach neighbourhood mostly in middle and low-income countries	VPD***	Living in difficult conditions	1) Many different factors associated with immunization status strongly varying by investigated area 2) Community involvement has shown to face several factors for low immunization at the same time 3) Physical distance to health services should be reduced 4) Maternal education has shown to be effective
Eiset et al. (2017) [5]	Review of infectious diseases in refugees and asylum seekers-current status and going forward	51	Refugees and other migrants	Infectious diseases	Migrant status (prevalence studies)	1) Prevalence of TB is rising 2) Infectious diseases are important in refugees 3) Risk of transmission to autochthonous population is low 4) Refugee status and context of flight is rarely considered in studies
Hvass et al. (2017) [22]	Systematic health screening of refugees after resettlement in recipient countries	53	Refugees	Screened infectious diseases	Implemented health screenings	1) Circumstances of screening strongly depend on recipient country 2) Most common screened diseases are TB, parasites, hepatitis and anaemia 3) Though important–mental health issues and chronic diseases were only screened in a few studies
Mipatrini et al. (2017) [9]	Vaccinations in migrants and refugees—a challenge for european health systems	58	Migrants and refugees in Europe	VPD***	Strategies for assessment and immunisation	1) Health systems of countries of origin often are disrupted from war, leading to risk of critical infection with VPD*** 2) Polio and MMR-vaccines should be prioritised, tetanus, diphtheria and hep. B. As well
Pavli et al. (2017) [4]	Health problems of newly arrived migrants and refugees in europe	n.i	Refugees and migrants in Europe	Infectious and other diseases	Migrant status (prevalence studies)	1) Prevalence and disease-spectrums vary by country of origin 2) Respiratory diseases are the most common health issue at the Greek-Turkish border 3) Access to health care is often influenced by legal limitations for refugees
Pottie et al. (2017) [80]	[Review-protocol] Prevention and assessment of infectious diseases among children and adult migrants arriving to the European Union/European Economic Association	n.a	Migrants in Europe	Tuberculosis Hepatitis b and c VPD*** HIV Intestinal parasites	Being targeted by any prevention and assessment strategy considered	Data not yet published-
Chernet et al. (2018) [81]	Prevalence rates of six selected infectious diseases among African migrants and refugees	113	Migrants/ refugees of African origin	Hepatitis b and c Intestinal parasites Syphilis	Migrant status (prevalence studies)	1) Blood-borne infections are more relevant in refugees than intestinal parasitic infections 2) Transmission cycle of parasitic infections is interrupted in recipient countries 3) Geographic region of origin shows correlation with disease-spectrum
Nellums et al. (2018) [6]	Antimicrobial resistance among migrants in Europe	23	Migrants in Europe	Infection with AMR°	Migrant status (observational studies)	1) Prevalence of AMR in migrants is about 25% overall 2) Prevalence of AMR higher in refugees/asylum seekers than other migrants 3) No data found on transmission to autochthonous population
Seedat et al. (2018) [23]	How effective are approaches to migrant screening for infectious diseases in Europe?	47	Migrants in Europe	Screened infectious diseases	Implemented health screenings	1) Innovative strategies should be implemented for completion of screening and treatment 2) Coverage of screening is low 3) EU/EEA approach of screening is too restrictive/focussed on single diseases

*(= radiological), cell-mediated, serological, microbiological, microscopical)

***vaccine preventable diseases

°antimicrobial resistant pathogens

The objective of this systematic review is to collect and synthesize evidence on the effect of interventions to diagnose, treat and manage respiratory-tract infections in refugees and immigrants in OECD, EU, EEA and EU-applicant countries.

## Methods

This review was carried out in accordance with the PRISMA Statement [36] and the Cochrane Handbook [37, 38]. (PROSPERO: CRD42018074338) [39]. A completed PRISMA checklist is provided as Additional file.

### Eligibility for inclusion

We included controlled studies (including non-randomised intervention studies and longitudinal studies with pre-test/post-test designs) published between 1 January 2000 and 1 October 2019. Study populations were refugees [40], asylum seekers [40] or immigrant hard-to-reach populations (e.g. Sinti and Roma, sex workers, drug abusers, prisoners, homeless people and people living with HIV [29, 41]). As previously mentioned, we use the term ‘’refugees and immigrants’’ whenever talking about this population in general. Place of intervention was a member country of the OECD [42], the European Union [43, 44], the European Economic Area (EEA) [45] or an European Union applicant country [46] (For full list of included countries see Additional file 1). Included were interventions to reduce incidence, prevalence, mortality, delay to diagnosis and treatment and transmission from respiratory infections, including lower and upper respiratory infections of unknown aetiology, acute respiratory infections and respiratory infections with pathogens listed in the PICOS table (Table 3). Also included were interventions to improve vaccination coverage for respiratory-related vaccine-preventable diseases. We excluded interventions to reduce tuberculosis incidence due to the existence of several recently-published systematic reviews [25–27, 29, 41]. Studies with any type of individual- or group-level interventions were included: outbreak prevention, any type of vaccination campaign, health literacy interventions, pharmaceutical and non-pharmaceutical prevention and treatment strategies, housing interventions and local health care arrangements.

**Table 3:** PICOS table

P	Refugees[40] Asylum seekers[40] Migrant hard-to-reach populations[41] Living in the geographical territory of OECD[42], EU[43], EEA[45] and EU-applicant [46] countries			
I	All interventions targeting to lower incidence, prevalence, transmission, or mortality of one or more of the following respiratory infections:			
	Upper respiratory infections	Lower respiratory infections	Included edge-cases	Separate pathogens
	Sinusitis Otitis Stomatitis (+Herpes) Catarrh Laryngitis Epiglottitis Scarlet fever Rhinoscleroma Nasopharyngitis Rhinitis (+Coryza) Diphtheria Tonsillitis Pharyngitis Supraglottitis	Pneumonia Pseudo-Croup Pleuritis Empyema Tracheitis Bronchitis Bronchiolitis (+obstr.) Bronchopneumonia Pleuropneumonia Pertussis	Abscesses: Mediastinal Peritonsillar Retropharyngeal Lung Influenza Mumps Measles Mononucleosis Chickenpox	Haemophilus Measles virus Influenza virus EBV RSV Mumps virus Varicella Adenovirus Pasteurella *C. diphtheriae* Pneumococcus Bordetella Legionellosis Aspergillosis Blastocysts Blastomycosis Histoplasmosis Mycoplasma
	These interventions include strategies for outbreak prevention, any type of vaccination campaign, local health care arrangements, individual or institutional health literacy interventions pharmaceutical and non-pharmaceutical prevention and treatment strategies and housing interventions			
C	Refugees and immigrants not (yet) having had access to the respective intervention			
O	Effect of intervention measured by change of: Incidence or prevalence of respiratory infections Mortality of respiratory infections Delay to diagnosis or delay to treatment of respiratory infections Transmission and secondary case-numbers of respiratory infections			
S	All controlled studies including studies with pre-test/post-test-design and non-randomised intervention studies			

Outcomes were change in diseases incidence, prevalence or mortality, delay to diagnosis or treatment, transmission rates, number of secondary cases and vaccination coverage.

### Search strategy, study selection and data collection

The database search was conducted following the Cochrane Collaboration guideline for systematic literature searches [47, 48]. The following databases were searched in June 2017. The search was updated in May 2018 and October 2019:

- MEDLINE® using OvidSP (including: Ovid MEDLINE® Epub Ahead of Print, In-Process & Non-Indexed Citations, Ovid MEDLINE® Daily)
- Web of Science by Clarivate Analytics using All Databases
- CINAHL using EBSCOhost
- PSYNDEX using EBSCOhost.

The search terms in the literature review included specific terms for respiratory infections, unspecific terms for infections and inflammation in general, terms for anatomic and topographic localisations, and terms for refugees and immigrants. The Medline® search was complemented by a Mesh Term search. The full search strategy can be found in Additional file 3.

References were screened by title and abstract by two reviewers (JFL and AB) independently, consensus was reached via consultation with a third reviewer (BL). For all selected abstracts full texts were obtained. We extracted information on study characteristics, study population, study design and outcome measures. In cases of incomplete or inconsistent data, authors were contacted.

Interrater agreement was calculated with kappa-statistics using the respective decisions whether to include or to exclude a study of the two reviewers J-F L and AB.

Our used data collection form is provided in Additional file 2.

After extraction, data was summarised and represented in tabular form by outcomes and outcome measures: Incidence rate, outbreak measures in number of secondary cases per exposed individual and vaccination coverage as percentage of being vaccinated. Further outcome measures were not found, i.e. mortality rate, delay to diagnosis and treatment. Whenever possible, we calculated risk or rate ratios comparing intervention and control groups to achieve better comparability.

### Risk of bias assessment

Risk of bias assessment was carried out using the ROBINS-I tool for cohort-style non-randomised trials of interventions for all included non-randomised trials [49] and using RoB2.0 for all included randomised controlled trials [50]. Risk of bias across studies was evaluated for publication and language bias and following the GRADE recommendations in the range of outcome subgroups across studies [51–55].

## Results

### Study Selection and characteristics

**Fig. 1:**
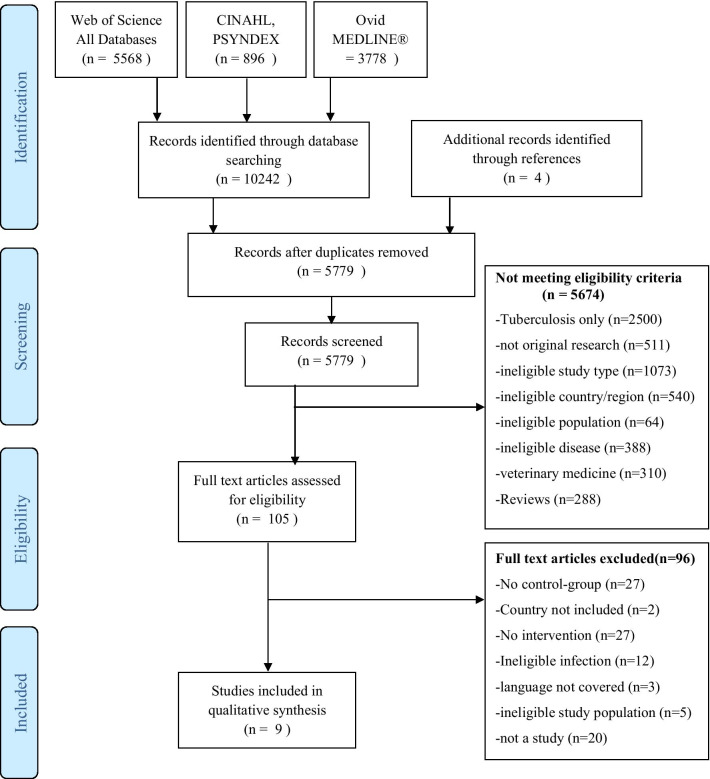
Prisma Flow-Chart (separately uploaded)

After manual deduplication 5779 of 10,242 abstracts remained for screening. After title and abstract screening 105 full texts were assessed of which 9 studies were included in the review. Inter-reviewer reliability was high. The calculated kappa was = 1. While the actual decisions whether to include or exclude a study were the same in 100% of cases (kappa = 1), only in 96% the reasons for these decisions were the same. The PRISMA flowchart is displayed in Fig. 1.

### Results of individual studies

(See Table 4 and 5).

**Table 4:** Characteristics of included studies

Author, country, year		Title	Study design	Type of intervention	Allocation of intervention	Outcome (measure)	Claimed vaccination strategy (if applicable)	Type of intervention and control group	N	Population included	Setting (refugee camp, city etc.)	% a&r	Mean age, % children	Country of origin/flight route
De vallière et al., Switzerland, 2011 [69]		Comparison of two strategies to prevent varicella outbreaks in housing facilities for asylum seekers	Non-randomised intervention study of cohort type	Vaccination of all individuals aged 15–39 years without contraindications	Allocated by date of arrival	Varicella outbreak-prevention (number of infected secondary cases)	All refugees without contraindication aged 15–39 years	Arrived 02/2009 to 05/2010	966	Recently arrived refugees	Refugee reception centre in Switzerland	100% asylum seekers	Unclear	Various countries
				Serotesting all exposed and vaccination of seronegative individuals			All exposed and tested seronegative	Arrived 05/2008 to 01/2009	858 (exposed: 248)					
				No outbreak-response-strategy			No response strategy	Arrived before 05/2008	126					
Pallasch et al., germany, 2005 [67]		Improvement of protection given by vaccination for socially underprivileged groups on the basis of "key persons approach"[German]	Non-randomised intervention study of cohort type	Door-to-door MMR-vaccination campaign based on “keyperson’’-principle	Allocated by residency	MMR-vaccination (children with at least one dose (%) and with two doses (%) of MMR-vaccine)	All children aged 15 months to 10 years	Children under 10 years (measured only school children at preterm examination: 39.8%)	344 (measured: 129)	Refugees and other inhabitants of hard-to-reach-neighbourhoods	German hard-to-reach and refugee neighbourhood ”Altländer-Viertel’’ in the city of Stade	About 30%	100% children under 10 years	Many diverse countries of origin
								Pre-intervention						
Brockman et al., germany, 2016 [68]		Ögd-initiative zur verbes-serung Der durch-impfung bei Asylsuch-enden	Non-randomised intervention study of cohort type	Housing based MMR and DPPT vaccination concept	Allocated by residency	Persons with at least one vaccination (%)	All asylum seekers in community housings	Community housing included in campaign	2256	Refugees and asylum seekers in community housings	German community housings for asylum seekers in the district of Reutlingen	100%	Unclear	Various countries
								Community housings excluded from campaign						
Koop et al., Macedonia, 2001 [66]		Results of the expanded program on immunization in the Macedonia refugee camps	Non-randomised intervention study of cohort type	Two consecutive vaccination campaigns for DPPT and BCG in stable and unstable population	No allocation, comparison of two refugee camps	Measles-, DPPT- and BCG -vaccination (vaccination coverage (%) after each campaign)	All children under 4 years of age (10% of overall study population)	Unstable population, high turnover	46,600 (included: 4660)	Albanian Kosovoar refugees	Macedonian refugee camp, “Brazda’’	100%	Included: 100% children aged under 4 years	Kosovo
								Stable population, low turnover	73,300 (included: 7330)		Macedonian refugee camp “Cegrane’’			
Coady et al., USA, 2008 [58]		A multilevel community-based intervention to increase influenza vaccination rates among hard-to-reach populations in New York city	Non-randomised intervention study of cohort type	Provision of informational materials concerning influenza on different levels and vaccination by nurses and physicians	Allocated by residency and revenue	Influenza vaccination (proportion of people with interest in getting vaccinated against influenza (%))	All residents with indication	Post-intervention	3082	Immigrant and hard-to-reach	Hard-to-reach neighbourhoods of east Harlem and the Bronx (New York)	Unclear	41 y.o	70% hispanic, 16% undocumented
								Pre-intervention	3744					
Hoppe et al., USA, 2011 [60]		Achieving high coverage of h1n1 influenza vaccine in an ethnically diverse obstetric population	Non-randomised intervention study of cohort type	Informational and vaccination campaign for influenza	Allocated by enrolment in the birth clinic	Influenza vaccination (vaccination coverage against influenza (%))	All pregnant women	Pregnant women at birth clinic in Seattle	157	Ethnically diverse pregnant women	Birth clinic in Seattle	Unclear	Unclear	Unclear
								Cited data (10 states from pregnancy Risk assessment monitoring system (prams))	6255	Pregnant women from cited data	Pregnant women in USA			Unclear
Rodriguez-Rieiro et al., Spain, 2011 [59]		Vaccination against 2008/2009 and 2009/2010 seasonal influenza in Spain	Non-randomised intervention study of cohort type	Spanish national influenza vaccination campaign	Allocated by residency	Influenza vaccination (vaccination coverage against influenza (%))	All residents with indication	Post-intervention (after 2009/2010)	51,666 (immigrants: 3426)	National cohort of Spanish citizens concerned by national influenza-vaccination campaign (with 6,6% immigrant population)	Spain	Unclear	Unclear	Unclear
								Pre-intervention (before 2009/2010)						
Larson et al., USA, 2009 [56]		Effect of intensive education on knowledge, attitudes, and practices regarding upper respiratory infections among urban Latinos	Non-randomised intervention study of cohort type	Door-to-door informational campaigns concerning (prevention from) influenza	Allocated by residency	Influenza vaccination (proportion of households with at least one vaccinated member against influenza (%))	All residents with indication	Post-intervention	422	Heads of hard-to-reach and immigrant households	Hard-to-reach-neighbourhood upper Manhattan (New York)	Unclear	Unclear	97% Latino ethnicity, 90% foreign-born
								Pre-intervention	422					
Larson et al., USA, 2010 [57]	Impact of non-pharmaceutical interventions on URIS and influenza in crowded, urban households		Randomised controlled trial	Door-to-door informational campaign and provision of face masks and/or alcohol based hand sanitiser	Allocated by residency, random sampling	Influenza vaccination, prevention of URI, ILI and influenza (vaccination coverage against influenza after intervention period (%), incidence of URI, ILI and influenza (n/1000 person-weeks) during intervention period	All residents with indication	Group e only provided with information material	904	Immigrant and hard-to-reach	Hard-to-reach-neighbourhood upper Manhattan (New York)	Unclear	Unclear	90% Latino ethnicity, 50% foreign-born
								Group 1 additionally provided with hand sanitiser	946					
								Group 2 additionally provided with face masks and hand sanitiser	938

**Table 5:** Results of included studies

	Intervention						Outcome				
Author (Year)	Type of intervention	Intervention groups		Vaccination		Allocation	Outcome	Outcome measure	Cohort results	Percentage (95% CI)	Relative Risks calculated according to absolutes given
De Vallière et al. (2011) [69]	Different response strategies to varicella outbreaks in refugee housing	No response-strategy		n.i		Time-span sampled	Varicella outbreak prevention	Number of secondary cases among refugees at same housing, attack rate (%)	16/126	12.8% (95% CI: 7.4–19.8%)	RR = 1 (control group)
		Serotesting refugees at same housing as outbreak		Seronegative tested refugees					7/248	2.8% (95% CI: 1.1–5.7%)	Getting infected: RR = 0.22 (95% CI: 0.09–0.53)
		General-vaccination		All refugees aged 15–39 without history					0/966	0% (95% CI: 0–0.38%)	Getting infected: RR = 0.004 (95% CI: 0.0002 to 0.066)
Pallasch et al. (2005) [67]	Door-to-door MMR vaccination campaign supported by keypersons targeting children aged from 15 months to 10 years	Before campaign (2002)		In all indicated cases following national standards		Pre-/post-evaluation	MMR vaccination	Vaccination-coverage (%) for at least one and two doses of vaccine respectively (results only measured in children during school preterm examinations)	21/38 at least one	55% (95% CI: 38.3–71.3%)	Having received after campaign At least one: RR = 1.51 (95% CI: 1.11–2.08) Two doses RR = 7.66 (95% CI: 2.52–23.3) Of vaccination
									3/38 two doses	8% (95% CI: 1.6–21.1%)	
		During campaign (2003)							34/48 at least one	70% (95% CI: 55.9–83.1%)	
									21/48 two doses	44% (95% CI: 29.5–58.8%)	
		After campaign (2004)							36/43 at least one	84% (95% CI: 69.3–93.1%)	
									26/43 two doses	60% (95% CI: 44.4–75.0%)	
Brock-Mann et al. (2016) [68]	Community housing based vaccination concept	Housing excluded from campaign		In all indicated cases following national standards		Sampled by residency	MMR and DPPT vaccination	Percentage of persons with at least one vaccination (%)	71/704	10% (95% CI: 7.9–12.7%)	RR = 1 (control group)
		Housing included in campaign							571/1552	36.8% (95% CI: 33.8–39.9%)	Getting vaccinated: RR = 3.65 (95% CI: 2.89–4.59)
Koop et al. (2001) [66]	Childhood vaccination campaigns in high vs. Low turn-over populations	Unstable population		All children under 4 years of age		Sampled by time and place of arrival	Measles, DPPT, BCG Vaccination	Change in vaccination coverage between first and second campaign in children under 4 years (10% of total study population)	From first campaign 2567/2760 to 1387/1900 after second campaign	-20% - From 93.01% (95% CI: 91.9%- 93.4%) to 73% (95% CI: 70.9–74.9%)	Being vaccinated RR = 0.79 (95% CI: 0.77–0.81)
		Stable population							From first campaign 2867/3150 to 3804/4180 after second campaign	Stable at about 91% (95% CI: 89.9–91.9%) to 91%	Being vaccinated RR = 1.02 (95% CI: 1.001–1.03)
Coady et al. (2008) [58]	Multilevel Influenza informational campaign	Before campaign		None		Pre-/post-evaluation	Influenza vaccination	Interest in vaccination against influenza (%)	2995/3744 (80%)	80% (95% CI: 78.7–81.3%)	Interest in vaccination RR = 1.175 (95% CI: 1.15–1.20)
		After informational campaign							2897/3082 (94%)	94% (95% CI: 93.1–94.8%)	
Hoppe et al. (2011) [60]	Influenza informational and vaccination campaign in birth clinic	After informational campaign		In all willing patients		Sampled by choice of birth clinic	Influenza vaccination	Influenza vaccination coverage (%)	120/157 (76%)	76% (95% CI: 69.0–82.8%)	Being vaccinated RR = 1.565 (95% CI: 1.43–1.71)
		Cited national data		N.i					5538/11337 (48.8%)	48,8% (95% CI: 47.9–49.7%)	
Rodriguez-Rieiro et al. (2011) [59]	National Influenza vaccination campaign	Before vaccination campaign		In all indicated cases following national standards		Pre-/post-evaluation	Influenza vaccination	Odds of getting influenza vaccination for immigrants; reported: 1.10 (95% CI: 0.97–1.25)	163/2055 (7.9%)	7,9% (95% CI: 6.8–9.2%)	Being vaccinated RR = 1.27 (95% CI: 1.02–1.58)
		After vaccination campaign							138/1371 (10%)	10% (95% CI: 8.5–11.8%)	
Larson et al. (2009) [56]	Door-to-door based Influenza informational campaign	Before informational campaign		None		Pre-/post-evaluation	Influenza vaccination	Reported households with at least one vaccinated person (%)	269/422 (63.7%)	63.7% (95% CI: 58.9–68.3%)	Having vaccinated person living in household RR = 1.16 (95% CI: 1.06–1.27)
		After campaign							312/422 (73.9%)	73.9% (95% CI: 69.5–78.1%)	
Larson et al. (2010) [57]	Door-to-door based influenza, influenza-like illness (= ILI) and upper respiratory infections (= URI) informational, prevention and campaign	Group E (informational campaign only)	Before		None	Randomly sampled by households	Influenza vaccination	Mean change in vaccination coverage pre-/post campaign (%)	191/904 (21.1%)	21.1% (95% CI: 18.5–23.9%)	RR = 1.93 (95% CI: 1.67–2.24)
			After						369/904 (40.8%)	40,8% (95% CI: 37.6–44.1%)	
		Group 1 (additionally hand sanitiser)	Before						180/946 (19%)	19% (95% CI: 16.6–21.7%)	RR = 3.0 (95% CI: 2.60–3.46)
			After						540 /946 (57.1%)	57.1% (95% CI: 53.9–60.3%)	
		Group 2 (additionally hand sanitiser and face masks)	Before						210/938 (22.4%)	22.4% (95% CI: 19.8–25.2%)	RR = 1.94 (95% CI: 1.69–2.23)
			After						408/938 (43.5%)	43.5% (95% CI: 40.3–46.7%)	
		Group E					Prevention of URI**, ILI*** and influenza	URI**-rate/1000 person-weeks	35.38	95% CI: 33.7–37.1	RR = 1 (control-group)
		Group 1							29.06	95% CI: 27.6–30.6	RR = 0.82 (95% CI: 0.76–0.88)
		Group 2							38.91	95% CI: 37.2–40.7	RR = 1.1 (95% CI: 1.03–1.18)
		Group E						ILI***-rate/1000 person-weeks	2.26	95% CI: 1.8–2.7	RR = 1 (control-group)
		Group 1							1.93	95% CI: 1.6–2.4	RR = 0.85 (95% CI: 0.64–1.14)
		Group 2							1.56	95% CI: 1.2–1.9	RR = 0.69 (95% CI: 0.51–0.93)
		Group E						Influenza-rate/1000 person-weeks	0.52	95% CI: 0.33–0.77	RR = 1 (control-group)
		Group 1							0.60	95% CI: 0.39–0.85	RR = 1.15 (95% CI: 0.65–2.07)
		Group 2							0.49	95% CI: 0.32–0.73	RR = 0.96 (95% CI: 0.52–1.75)

**URI = Upper respiratory infection

***ILI = Influenza-like illness

While using the term ‘’refugees and immigrants’’ whenever talking in general about the targeted populations in this review we used the respective expression of the respective study when narrating the individual results.

### Influenza vaccination/interest in vaccination informational campaigns

Three studies assessed influenza vaccination in hard-to-reach neighbourhoods in New York City with an immigrant proportion of more than 70% [56–58]. Each evaluated multilingual information campaigns in various forms such as comic strips, informational talks, and community events responding to common myths about vaccinations and advising on locations of free vaccination clinics. Three different outcomes have been measured. The interest in getting vaccinated against influenza [58] increased by 14% post-intervention compared to pre-intervention. The probability of a person vaccinated against influenza living in the same household as the respondent [56], increased by 10.2% pre-intervention compared to post-intervention. A randomised controlled study compared health literacy campaign with added non-pharmaceutical prevention strategies against influenza and upper respiratory infections [57]: one group (Group E) received only the informational campaign, another (Group 1) received additional alcohol-based hand sanitiser, and a third group (Group 2) received additional hand sanitiser and face masks. Influenza vaccination coverage improved in all groups after the intervention period by 19.7% in Group E, by 38.1% in Group 1 and by 21.1% in Group 2.

One study retrospectively evaluated the effect of the national vaccination campaigns against influenza in Spain in 2008/2009 and 2009/2010 [59]. There was no evidence of an increase in vaccination coverage in immigrants; the proportion of vaccinated immigrants increased insignificantly by 2.1% compared to the years before. We were unable to identify the main content of the campaign interventions. We found no official records of the interventions and received no answer to our requests for information.

In pregnant women of varying ethnicities in Seattle [60], influenza vaccination coverage increased by 27.2% post-intervention, which compares to data from the Pregnancy Risk Assessment Monitoring System (PRAMS) from separate studies [61–65]. The intervention in this study comprised three months of multi-language information and video material in the waiting room of a birth-clinic as well as personal talks and “flu-packs” consisting of face masks, hand sanitiser and thermometers [60].

### Vaccination campaigns for respiratory tract-related childhood diseases

Three studies evaluated different vaccination campaigns for respiratory tract-related childhood diseases [66–68]. One compared the effectiveness of two consecutive mass vaccination campaigns of governmental and non-governmental organisations against 8 antigens in recently-arrived refugee children under 4 years of age fleeing from the Kosovo Crisis (1998/1999) in Macedonian refugee camps with high and low turnover [66], showing a benefit for more stable populations with less turnover: in a camp with high population turnover vaccination coverage rates dropped by 20% after the second campaign three weeks later, while in a stable population camp vaccination coverage stayed constant after both campaigns. Before the second mass vaccination weekly vaccination clinics were initiated in the respective camps.

In a second study the Ministry of Health in German reception centres implemented a vaccination campaign for MMR and DPPT which involved vaccine doses, informational material and interpreters to ensure informed consent in newly arrived refugees. The percentage of refugees with at least one vaccination increased by 26.8% compared to pre-intervention.

The third study evaluated the effect of the “keyperson’’ principle enhancing the effectiveness of a MMR vaccination campaign for children under 10 years of age [67]. *Keypersons* are defined as trained persons from similar living conditions and ethnicities. In this study, they assisted in a door-to-door vaccination campaign in German hard-to-reach and broad-spectrum immigrant neighbourhoods with refugee proportions of about 30%. The risk of receiving at least one dose of vaccination increased by 29%, while the risk of receiving two doses increased by 52%.

### Outbreak prevention

In a Swiss interventional study, two varicella outbreak response strategies were evaluated in newly arrived refugees [69]. The first (”rapid’’) involved the isolation of index cases and serotesting and, if necessary, vaccination of people in the same housing facility. This was found to lower the proportion of infected persons from by 10% compared to an outbreak series in 2007 with no previously defined response strategy. The second (“general’’) response strategy involved the strict vaccination of all arriving refugees aged 15–39 years without a history of varicella, leading to no varicella infections in any refugee shelter.

### URI/ILI/Influenza prevention

The previously-described randomised controlled trial by Larson et al. in hard-to-reach neighbourhoods also assessed rates of upper respiratory infections (URI), influenza and influenza-like illness (ILI) [57]. It was found that the URI rate/1000 person-weeks in the control group E (informational campaign only) was 35.38, in Group 1 (additional hand sanitiser) it was 29.06 and in Group 2 (hand sanitiser and face masks) it was 38.91. ILI rates/1000 person-weeks were 2.26 in Group E, 1.93 in Group 1, and 1.56 in Group 2. Influenza rates/1000 person-weeks were 0.52 in Group E, 0.60 in Group 1, and 0.49, 95% CI: 0.32–0.73 in Group 2.

### Main evidence gaps

The main evidence gaps include a lack of data on housing arrangement standards, interventions to reduce rates of bacterial pneumonia, and evidence for the efficacy of operating standards in refugee and immigrant health care and reception centres (Table 6).

**Table 6:** Evidence gaps identified in this systematic review

_Outcome_	_Type of intervention_	_TB_	_Influenza_	_Bacterial Pneumonia and lower respiratory infections_	_Other_
_Reducing morbidity or mortality_	_Information campaigns_	_+_	−	−	−
	_Housing standards_	−	−	−	−
	_Health literacy campaigns_	_+_	−	−	−
	_Operational standards for health care_	−	−	−	−
	_Personal Hygiene_	_+_	_+_	−	_+_
_Increasing vaccination coverage_	_Information campaigns_	_+_	_+_	−	_+_
	_Housing standards_	_-_	_-_	−	−
	_Health literacy campaigns_	_+_	_+_	−	−
	_Operational standards for health care_	−	−	−	−

### Risk of bias

(See Table 7).

**Table 7:** Risk of bias assessment of included studies

	Using ROBINS-I-tool for non-randomised studies of cohort-type // Risk of bias due to:							
Author (year of publication)	Confounding	Selection of participants	Classification of interventions	Deviation from intended interventions	Missing data	Measurement of outcomes	Selection of reported result	Overall judgement
De Vallière et al. (2011) [69]	Low	Low	Low	Low	Low	Low	Low	Low
Pallasch et al. (2005) [67]	Moderate	Moderate	Low	Low	Moderate	Moderate	Moderate	Moderate
Brockmann et al. (2016) [68]	Serious	Moderate	Low	Low	Moderate	Moderate	Serious	Serious
Koop et al. (2001) [66]	Serious	Low	Moderate	Moderate	Low	Low	Serious	Serious
Coady et al. (2008) [58]	Moderate	Low	Low	Low	Low	Moderate	Low	Moderate
Hoppe et al. (2011) [60]	Serious	Serious	Low	Low	Critical	Serious	Serious	Critical
Rodriguez-Rieiro et al. (2011) [59]	Critical	Critical	Critical	Serious	Moderate	Moderate	Critical	Critical
Larson et al. (2009) [56]	Serious	Low	Moderate	Low	Serious	Serious	Serious	Serious
	Using RoB 2.0 for cluster-randomised parallel group trials of intervention // Risk of bias due to							
	Randomisation process	Timing of identification and recruitment of individual participants in relation to timing of randomisation		Deviations from intended interventions	Missing outcome data	Measurement of outcomes	Selection of reported result	Overall judgement
Larson et al. (2010) [57]	Low [O1-O4]	Low [O1-O4]		Some concerns [O1-O4]	Low [O1-O4]	Some concerns [O1-O4]	Low [O1-O4]	Some concerns [O1-O4]

The most important sources of bias among studies targeting vaccination coverage of respiratory tract-related infections were confounding bias in seven [56, 58–60, 66, 68, 70], reporting bias in six [56, 59, 60, 66, 68, 70] and bias due to the measurement of outcomes in 6 studies [56, 59, 60, 66, 68, 70]. The most important non reported confounding factors were length of travel in seven [56–60, 66, 68–70], period of stay in seven [56–60, 66, 70] and health literacy in seven studies [56, 58–60, 66–68]. The main source of bias among studies targeting prevention of upper respiratory tract infections, influenza-like illness, influenza and vaccination coverage for influenza was social desirability bias in three studies [56–58]. In two studies the risk of bias was rated as critical; one because of missing data [60], and the other mainly due to incorrect classification of interventions [59].

Across studies the heterogeneous outcome measures could be an important source of bias, especially among the studies on influenza vaccination uptake, as interest in being vaccinated [58] or the presence of a vaccinated person in a household [56] may not be valid measures of actual vaccination coverage [57, 60] in the examined population. Publication bias may also be an important source of bias, because with only two exceptions [57, 66], no negative intervention effect was described. Therefore, the findings regarding the overall effect of these interventions should be interpreted with caution [51] and evidence across all outcomes is evaluated as “low’’ following the GRADE approach.

Since the included studies have heterogeneous study designs, characteristics and outcome measures, no pooled analysis in the form of a meta-analysis was performed.

## Discussion

### Key results

In this systematic review of controlled studies, we collected evidence on interventions aiming to reduce morbidity or mortality from respiratory infections and to improve vaccination coverage among refugees and immigrants in hard-to-reach neighbourhoods. In six studies, we found evidence for the effectiveness of multilingual informational campaigns to increase the uptake of vaccination [56–58, 60, 67, 68].

There was limited evidence in one study that hand sanitiser reduced rates of upper respiratory infections and when provided together with face masks also the rates of influenza-like-illness in a hard to reach migrant neighbourhood [57].

To reduce secondary cases in outbreak situations, one study reported that in the case of vaccine-preventable diseases, general vaccination strategies implemented in refugees immediately after arrival have a large effect in reducing secondary cases. Evaluation of a positive history of infection or vaccination is sufficient, but serological testing after potential exposure is not necessary [69]. The hazard of outbreak situations is also lowered by this strategy. [69]

### Evidence rarely reflected in international standards

Five informational campaigns assessed in this review integrated the cultural and educational background of the targeted population [56–58, 60, 67], either by recruiting *keypersons* supporting the campaign staff [67], or by directly addressing common cultural myths about vaccinations [57, 58]. The efficacy of this strategy is not currently reflected in national or international guidelines, but is congruent with evidence that informational campaigns, community und culturally-oriented health literacy interventions are effective in improving vaccination coverage in autochthonous populations [71–73].

Currently, only NICE recommends institutional health literacy interventions in immigrant populations, however these are not clearly defined. Other European guidelines for refugee and immigrant health care do not address this issue (Table 1).

European guidelines explicitly recommend against implementation of vaccination strategies at border crossings because informed consent is difficult to obtain [15]. However, a timely vaccination strategy has been shown to reduce the potential for outbreaks in particular for highly transmissible diseases [69]. Research is needed to determine the optimal strategies for obtaining informed consent in this situation [74–76].

No evidence could be found for interventions targeting housing facilities for refugees and immigrants, although they play an important role in the transmission and spreading of infectious diseases [15]. The effect of turn-over of refugees and immigrants on vaccination coverage in refugee camps shows that it is easier to implement vaccination campaigns during the first months after arrival in more stable camps, but this study did not investigate overall housing conditions or number of housing units [66]. We did not find any controlled studies of interventions to reduce morbidity and mortality from relevant severe respiratory diseases such as bacterial pneumonia or interventions affecting the latency to diagnosis in airway infections.

## Limitations

Migration status was not always clear for all participants in the identified studies. While most of the studies indicated the ethnic or geographical origin of the participants [56–58, 60, 67], only two primarily represented refugee populations [66, 69]. However, interventions in the context of having fled from hardship, forced migration and life as a refugee are possibly presenting an important independent factor [5]. Countries of origin were not always clearly indicated, especially for Latin American and African populations. In addition, the heterogeneous outcome measures among the included studies made it difficult to obtain concrete effect estimates for refugee and immigrant populations. Some of the findings should be interpreted with caution due to methodological issues in several of the studies (see Table 7).

The countries of origin of refugees and immigrants have shifted in the last 10 years [77, 78], therefore we included a longer time-span to provide a broader overview and context in relation to refugee health.

## Conclusions

Respiratory infections continue to be a major contributor to the poor health status of refugees and immigrants and currently interventions to lower this burden are not adequately reflected in national guidelines. This evidence synthesis shows that while there is some low-quality evidence for the effect and timing of multilingual vaccination campaigns that involve the community, there is little high-quality research on housing standards or operational standards needed to prevent respiratory infections in this population. Similar to other reviews of refugee and hard-to-reach populations for tuberculosis [29, 41], vaccine-preventable diseases [79] and respiratory infections [8], we conclude that it is important to follow community-involving principles in informational campaigns and that more controlled operational research is needed. New methods of ensuring informed consent for early vaccinations must also be established. This could allow immediate vaccinations upon arrival in order to reduce secondary infections in crowded living portals such as reception centres and mass housings. Also, for future research it is essential to indicate the political status of migrant study populations because the context of forced migration may present an important independent factor.

## Supplementary Information

**Additional file 1.** Included countries.

**Additional file 2.** Data extraction spreadsheet.

**Additional file 3.** Full search strategy.

## Data Availability

Not applicable. Full search strategy in Additional file 3. Data extraction spreadsheet in Additional file 2. Our full extraction dataset will only be available on request.

## Abbreviations

Tb: Tuberculosis
VPD: Vaccine preventable diseases
AMR: Antimicrobial resistant pathogens
OECD: Organisation for Economic Co-operation and Development
EU: European Union
EEA: European Economic Area
ILI: Influenza-like-illness
URI: Upper respiratory infections
