# Supplementary material for: Reducing burden from respiratory infections in refugees and immigrants: a systematic review of interventions in OECD, EU, EEA and EU-applicant countries
Source: BMC Infect Dis. 2021 Aug 26;21:872. doi: 10.1186/s12879-021-06474-0 (PMC8390210; doi:10.1186/s12879-021-06474-0)
Supplement: Supplementary file 1 — Additional file 1. Included countries. [file 12879_2021_6474_MOESM1_ESM.docx]

| **European Union** | **EU-applicants** | **EEA-countries additional** | **OECD-** |
| --- | --- | --- | --- |
| [Belgiu](https://de.wikipedia.org/wiki/Belgien)m | [Albania](https://de.wikipedia.org/wiki/Albanien) | Iceland | Australia |
| [Germany](https://de.wikipedia.org/wiki/Deutschland) | [Macedoni](https://de.wikipedia.org/wiki/Mazedonien)a | Norway | Chile |
| [France](https://de.wikipedia.org/wiki/Frankreich) | [Montenegro](https://de.wikipedia.org/wiki/Montenegro) | Liechtenstein | Israel |
| [Ital](https://de.wikipedia.org/wiki/Italien)y | [Serbi](https://de.wikipedia.org/wiki/Serbien)a |  | Japan |
| [Luxemburg](https://de.wikipedia.org/wiki/Luxemburg) | [Turke](https://de.wikipedia.org/wiki/T%C3%BCrkei)y |  | Canada |
| [Netherlands](https://de.wikipedia.org/wiki/Niederlande) |  |  | South Korea |
| [Denmark](https://de.wikipedia.org/wiki/D%C3%A4nemark) |  |  | Mexico |
| [Ireland](https://de.wikipedia.org/wiki/Irland) |  |  | New Zealand |
| [United](https://de.wikipedia.org/wiki/Vereinigtes_K%C3%B6nigreich) Kingdom |  |  | Switzerland |
| [Greece](https://de.wikipedia.org/wiki/Griechenland) |  |  | USA |
| [Portugal](https://de.wikipedia.org/wiki/Portugal) |  |  |  |
| [Spain](https://de.wikipedia.org/wiki/Spanien) |  |  |  |
| [Finland](https://de.wikipedia.org/wiki/Finnland) |  |  |  |
| [Austria](https://de.wikipedia.org/wiki/%C3%96sterreich) |  |  |  |
| [Sweden](https://de.wikipedia.org/wiki/Schweden) |  |  |  |
| [Estonia](https://de.wikipedia.org/wiki/Estland) |  |  |  |
| [Latvia](https://de.wikipedia.org/wiki/Lettland) |  |  |  |
| [Lithuania](https://de.wikipedia.org/wiki/Litauen) |  |  |  |
| [Malta](https://de.wikipedia.org/wiki/Malta) |  |  |  |
| [Polan](https://de.wikipedia.org/wiki/Polen)d |  |  |  |
| [Slovakia](https://de.wikipedia.org/wiki/Slowakei) |  |  |  |
| [Sloveni](https://de.wikipedia.org/wiki/Slowenien)a |  |  |  |
| Czech Republic |  |  |  |
| [Hungary](https://de.wikipedia.org/wiki/Ungarn) |  |  |  |
| [Cyprus](https://de.wikipedia.org/wiki/Republik_Zypern) |  |  |  |
| [Bulgar](https://de.wikipedia.org/wiki/Bulgarien)ia |  |  |  |
| [Romania](https://de.wikipedia.org/wiki/Rum%C3%A4nien) |  |  |  |
| [Croati](https://de.wikipedia.org/wiki/Kroatien)a |  |  |  |
