# Supplementary material for: Reducing burden from respiratory infections in refugees and immigrants: a systematic review of interventions in OECD, EU, EEA and EU-applicant countries
Source: BMC Infect Dis. 2021 Aug 26;21:872. doi: 10.1186/s12879-021-06474-0 (PMC8390210; doi:10.1186/s12879-021-06474-0)
Supplement: Supplementary file 2 — Additional file 2. Data extraction spreadsheet. [file 12879_2021_6474_MOESM2_ESM.pdf]

Study ID		Topic/Infection				
	-					
**Author**	**Studyname**	**Year of Study**	**Name of Infection/Complication**	**Kind of Study**	**Timespan**	**Country of realization**

Study Characteristics

Study Design

Individuals (number)	Loss of follow-up (number)	Sampling?	Name/Kind	Number	Females (%)	Country of Origin

PICO

Population			*Intervention*	
**Destination Country**	**Time since arrival** *(weeks/days/months/years?)*	**Loss of follow-up?**	**Name/Kind**	**Name/Kind**

						Results
						Effect
Comparison			*Outcome*		*Quality*	
**Number + Part of Refugees/A-S (%)**	**Females (%)**	**Loss of follow-up?**	**Name/Kind**	**Effective? (yes/no)**	**Description of Effect**	

		Reliability		Publication		
Quantity						
**(RR/OR/%/?)**	**CI / standard deviation**	**Limitations**	**Year of Publication**	**Journal/Source**	**Database online**	**D[illegible]**

Robins-I -Evaluation

Problems for inclusion

Outcome (several Outcomes: O1 =Outcome 1, O2 = Outcome 2, etc.)

ROB-Level

OI	Low	Moderate	Serious	Critical
