# Supplementary material for: Reducing burden from respiratory infections in refugees and immigrants: a systematic review of interventions in OECD, EU, EEA and EU-applicant countries
Source: BMC Infect Dis. 2021 Aug 26;21:872. doi: 10.1186/s12879-021-06474-0 (PMC8390210; doi:10.1186/s12879-021-06474-0)
Supplement: Supplementary file 3 — Additional file 3. Full search strategy. [file 12879_2021_6474_MOESM3_ESM.docx]

# Search Strategy

The search strategy included: a specific term for respiratory infections (Infec-SP), an unspecific term for infections and inflammations in general (Infec-NSP), a term for anatomic and topographic localisations (Locus) and a term for refugees, asylees and migrants (Population). Terms were combined using the following schedule:

**[(Infec-SP) OR ((Infec-NSP) AND* (Locus))] AND (Population)**

*****instead of ,,AND‘‘ in this case we used word-surrounding terms varying by search-surface (Ovid: Adj5, Web of Science: NEAR/5, EBSCOhost: N5)

We did not use any search terms specifying included countries or interventions to keep the search strategy as broad as possible.

The MeSH Terms used are marked in ‘’.’’ in the following search formula.

## Web of Science

## **( (** (tuberculo*) OR (TB) OR (LTBI) OR (latent* NEAR/2 tubercul*) OR (silicotubercul*) OR (Pneumon*) OR (Absce* NEAR/2 mediastin*) OR (Absce* NEAR/2 peritonsill*) OR (Absce* NEAR/2 retropharyngeal*) OR (Absce* NEAR/3 lung) OR (sinus*) OR (diphter*) OR (measle*) OR (Haemophil*) OR (whooping) OR (Pertussis) OR (parapertussis) OR (Mump*) OR (bronchitis) OR (bronchiolitis) OR (tonsillitis) OR (Pharyngit*) OR (tracheit*) OR (laryngit*) OR (laryngotracheit*) OR (Pseudocroup) OR (pseudo-croup) OR (croup) OR (croup-syndrom) OR (Krupp) OR (Pseudo-Krupp) OR (Epiglottit*) OR (pleuri*) OR (empye*) OR (otit*) OR (rhinit*) OR (legionn*) OR (Influenza) OR (Herpes*) OR (HSV) OR (RSV) OR (Human NEAR/2 Respiratory NEAR/2 Syncytial) OR (coryza) OR (scarlet) OR (aspergill*) OR (histoplasm*) OR (blastocyst*) OR (blastomyc*) OR (pasteur*) OR (Catarrh*) OR (influenza*) OR (flu) OR (mycoplasm*) OR (mononucleos*) OR (EBV) OR (Eppstein-Barr-) OR (glandular NEAR/2 fever) OR (Varicella) OR (*pox) OR (Stomatitis) OR (pneumococc*) OR (Pneumokokk*) OR (strept* NEAR/2 pneumon*) OR (Bordetell*) OR (Bronchopneumon*) OR (Pleuropneumon*) OR (Rhinosclero*) OR (Rhinosklero*) OR (Supraglottit*) OR (nasopharyngit*) OR (Common NEAR/2 Cold) OR (Adenovir*) OR (Adeno-vir*)

## **) OR ( (** (Respira*) OR (airway) OR (aerosol) OR (pulmon*) **) NEAR/5 (** (*itis) OR (*phyt) OR (*osis) OR (*iasis) OR (streptococc*) OR (bacteraem*) OR (viraem*) OR (viral*) OR (virus) OR (bacteri*) OR (septic*) OR (fever) OR (Chlamydia) OR (Ricketts*) OR (purulen*) OR (pus*) OR (spore*) OR (infec*) OR (protozo*) OR (Helmint*) OR (parasit*) OR (coxiell*) OR (vaccin*) **) ) ) AND (** (Refuge*) OR (migrant*) OR (asylum*) OR (immigrant*) OR (displaced NEAR/2 person*) **)**

## OVID

## **(**

## **(**

## (tuberculo*.mp.) OR (TB.mp.) OR (LTBI.mp.) OR (latent*.mp. ADJ2 tubercul*.mp.) OR

## (silicotubercul*.mp.) OR (Pneumon*.mp.) OR (Absce*.mp. ADJ2 mediastin*.mp.) OR

## (Absce*.mp. ADJ2 peritonsill*.mp.) OR (Absce*.mp. ADJ2 retropharyngeal*.mp.) OR

## (Absce*.mp. ADJ3 lung.mp.) OR (sinus*.mp.) OR (diphter*.mp.) OR (measle*.mp.) OR

## (Haemophil*.mp.) OR (whooping.mp.) OR (Pertussis.mp.) OR (parapertussis.mp.) OR

## (Mump*.mp.) OR (bronchitis.mp.) OR (bronchiolitis.mp.) OR (tonsillitis.mp.) OR

## (Pharyngit*.mp.) OR (tracheit*.mp.) OR (laryngit*.mp.) OR (laryngotracheit*.mp.) OR

## (Pseudocroup.mp.) OR (pseudo-croup.mp.) OR (croup.mp.) OR

## (croup-syndrom.mp.) OR (Krupp.mp.) OR (Pseudo-Krupp.mp.) OR (Epiglottit*.mp.) OR

## (pleuri*.mp.) OR (empye*.mp.) OR (otit*.mp.) OR (rhinit*.mp.) OR (legionn*.mp.) OR

## (Influenza.mp.) OR (Herpes*.mp.) OR (HSV.mp.) OR (RSV.mp.) OR

## (Human.mp. ADJ2 Respiratory.mp. ADJ2 Syncytial.mp.) OR (coryza.mp.) OR

## (scarlet.mp.) OR (aspergill*.mp.) OR (histoplasm*.mp.) OR (blastocyst*.mp.) OR

## (blastomyc*.mp.) OR (pasteur*.mp.) OR (Catarrh*.mp.) OR (influenza*.mp.) OR

## (flu.mp.) OR (mycoplasm*.mp.) OR (mononucleos*.mp.) OR (EBV.mp.) OR

## (Eppstein-Barr*.mp.) OR (glandular.mp. ADJ2 fever.mp.) OR

## (Varicella.mp.) OR (chickenpox.mp.) OR (Stomatitis.mp.) OR (pneumococc*.mp.) OR

## (Pneumokokk*.mp.) OR (strept*.mp. ADJ2 pneumon*.mp.) OR (Bordetell*.mp.) OR

## (Bronchopneumon*.mp.) OR (Pleuropneumon*.mp.) OR (Rhinosclero*.mp.) OR

## (Rhinosklero*.mp.) OR (Supraglottit*.mp.) OR (nasopharyngit*.mp.) OR

## (Common.mp. ADJ2 Cold.mp.) OR (Adenovir*.mp.) OR (Adeno-vir*.mp.)

## **)**

## **OR**

## **(**

## **(**

## (Respira*.mp.) OR (airway.mp.) OR (aerosol.mp.) OR (pulmon*.mp.)

## **)**

## **ADJ5**

## **(**

## (streptococc*.mp.) OR

## (bacteraem*.mp.) OR (viraem*.mp.) OR (viral*.mp.) OR (virus.mp.) OR (bacteri*.mp.) OR

## (septic*.mp.) OR (fever.mp.) OR (Chlamydia.mp.) OR

## (Ricketts*.mp.) OR (purulen*.mp.) OR (pus*.mp.) OR (spore*.mp.) OR (infec*.mp.) OR

## (protozo*.mp.) OR (Helmint*.mp.) OR (parasit*.mp.) OR (coxiell*.mp.) OR

## (vaccin*.mp.)

## **)**

## **)**

## **OR**

## **(**

## "Respiratory Tract Infections"/ OR

## "Otitis"/ OR "Labyrinthitis"/ OR "Otitis Externa"/ OR

## "Otitis Media"/ OR "Mastoiditis"/ OR

## "Otitis Media with Effusion"/ OR "Otitis Media, Suppurative"/ OR

## "Petrositis"/ OR "Stomatitis"/ OR

## "Stevens-Johnson Syndrome"/ OR "Stomatitis, Aphthous"/ OR

## "Stomatitis, Herpetic"/ OR "Vesicular Stomatitis"/ OR

## "Candidiasis, Oral"/ OR "Scarlet Fever"/ OR

## "Mumps"/ OR "Measles-Mumps-Rubella Vaccine"/ OR

## "Measles"/ OR "Subacute Sclerosing Panencephalitis"/ OR

## "Chickenpox"/ OR "Chickenpox Vaccine"/ OR

## "Encephalitis, Varicella Zoster"/ OR

## "Epstein-Barr Virus Infections"/ OR "Infectious Mononucleosis"/ OR

## "Respiratory Syncytial Virus Vaccines"/ OR

## "Pneumococcal Vaccines"/ OR

## "Heptavalent Pneumococcal Conjugate Vaccine"/ OR

## "Influenza Vaccines"/ OR "Adenovirus Vaccines"/ OR "Adenovirus Infections, Human"/ **) ) AND ( (**

## (Refuge*.mp.) OR (migrant*.mp.) OR (asylum*.mp.) OR (immigrant*.mp.) OR (displaced.mp. ADJ2 person*.mp.) **) OR (**

## "Refugees"/

## **) )**

## CINAHL

## **((**(tuberculo*) OR (TB) OR (LTBI) OR (latent* N2 tubercul*) OR (silicotubercul*) OR (Pneumon*) OR (Absce* N2 mediastin*) OR (Absce* N2 peritonsill*) OR (Absce* N2 retropharyngeal*) OR (Absce* N3 lung) OR (sinus*) OR (diphter*) OR (measle*) OR (Haemophil*) OR (whooping) OR (Pertussis) OR (parapertussis) OR (Mump*) OR (bronchitis) OR (bronchiolitis) OR (tonsillitis) OR (Pharyngit*) OR (tracheit*) OR (laryngit*) OR (laryngotracheit*) OR (Pseudocroup) OR (pseudo-croup) OR (croup) OR (croup-syndrom) OR (Krupp) OR (Pseudo-Krupp) OR (Epiglottit*) OR (pleuri*) OR (empye*) OR (otit*) OR (rhinit*) OR (legionn*) OR (Influenza) OR (Herpes*) OR (HSV) OR (RSV) OR (Human N2 Respiratory N2 Syncytial) OR (coryza) OR (scarlet) OR (aspergill*) OR (histoplasm*) OR (blastocyst*) OR (blastomyc*) OR (pasteur*) OR (Catarrh*) OR (influenza*) OR (flu) OR (mycoplasm*) OR (mononucleos*) OR (EBV) OR (Eppstein-Barr-) OR (glandular N2 fever) OR (Varicella) OR (*pox) OR (Stomatitis) OR (pneumococc*) OR (Pneumokokk*) OR (strept* N2 pneumon*) OR (Bordetell*) OR (Bronchopneumon*) OR (Pleuropneumon*) OR (Rhinosclero*) OR (Rhinosklero*) OR (Supraglottit*) OR (nasopharyngit*) OR (Common N2 Cold) OR (Adenovir*) OR (Adeno-vir*)**) OR (**(Respira*) OR (airway) OR (aerosol) OR (pulmon*)**) N5 (**(*itis) OR (*phyt) OR (*osis) OR (*iasis) OR (streptococc*) OR (bacteraem*) OR (viraem*) OR (viral*) OR (virus) OR (bacteri*) OR (septic*) OR (fever) OR (Chlamydia) OR (Ricketts*) OR (purulen*) OR (pus*) OR (spore*) OR (infec*) OR (protozo*) OR (Helmint*) OR (parasit*) OR (coxiell*) OR (vaccin*)**)) AND (**(Refuge*) OR (migrant*) OR (asylum*) OR (immigrant*) OR (displaced N2 person*)**)**
